# Future Time Orientation and Learning Engagement Through the Lens of Self-Determination Theory for Freshman: Evidence From Cross-Lagged Analysis

**DOI:** 10.3389/fpsyg.2021.760212

**Published:** 2022-03-10

**Authors:** Michael Yao-Ping Peng, Zizai Zhang

**Affiliations:** ^1^School of Economics and Management, Foshan University, Foshan, China; ^2^Hangzhou Preschool Teachers College, Zhejiang Normal University, Hangzhou, China

**Keywords:** cross-lagged analysis, future time orientation, learning engagement, self-determination theory, learning motivation

## Abstract

View of future time orientation is a cognitive construct about future time. This view has its unique work of motivation and effect on academic performance. Previous studies have only explored the influence that future time orientation brings to the learning process at a single time, and most of them focus on cross-sectional studies. To further explore the cross-lagged relationship for freshmen between future time orientation and learning engagement during different periods, AMOS 23.0 was performed for cross-lagged analysis in this study to explore the influence and effect among variables of different periods. This research was based on the theory of self-determination to discover the relationship between future time and learning involvement for freshmen in enrollment and the first summer vacation. In this research, there were 1,000 valid samples in the first stage and 840 valid samples in the second stage for the conduction of descriptive statistics, pair *t*-test, and cross-lagged analysis. The results show that: (1) for learning engagement, freshmen at the end of the first year have a higher average score than at the beginning of the first year. (2) View of students of future time orientation can affect their learning engagement of the future through self-determination of students. At last, we provide some suggestions as references for institutional research and future research.

## Introduction

In the United States, if freshmen can adapt well to the study or campus life in the first year of college, most of them can successfully complete the 4-year study, but if they fail to adapt to college life, most of them will leave campus in the first year (DeBerard et al., 2004). Considering the importance of the first year, many universities have launched learning interest orientation programs to help freshmen to adapt to the study or campus life in the first year (Visser and Hirsch, 2014). Nevertheless, since there are differences between Eastern and Western cultures, students in Asia will indeed face the life lessons and challenges of individual separation and individualization when they are in college (Zhang et al., 2015; Wong et al., 2019). Due to the changes in the college life environment, supervision of study and life from parents and other family members is becoming less, while more freedom, independence, and autonomous space are available for them. The self-determination theory provided that the social environment does not hinder the three basic psychological needs (De Bilde et al., 2011; Guay and Bureau, 2018), which are autonomy, ability, and relationship, the individual will produce intrinsic motivation and make people achieve self-fulfillment (Howard et al., 2017; Ryan and Deci, 2017, 2020); and in the process of self-fulfilling, there are expectations for possible life path in the future (De Bilde et al., 2011). However, since there are cultural differences, the theoretical verification results obtained from the self-determination theory in western society may not be the same in eastern society (Zhang et al., 2015; Lindstrom-Johnson et al., 2016). Thereby, this study is aimed to explore the future time orientation from freshmen in Asia from the perspective of self-determination.

According to the Expected Value Theory (Wigfield and Eccles, 2002; Wigfield et al., 2017), the expectation for success belongs to a kind of future orientation. When an individual holds a high expectation for the future, it will lead to higher persistence, learning behaviors, and higher self-motivation performance (Levitt et al., 2016). As for Chinese education, it starts from future time orientation, and learners usually base their learning goals on long-term future achievements (Zhang et al., 2015; Wong et al., 2019). The future time orientation concerned in this study indicates that people have positive performance in their personality and make plans for their own life (De Bilde et al., 2011). Gjesme (1983) once compared future time orientation to search light, which means that the better future time orientation of an individual is, the more it can illuminate the way forward, the further results can help the individual to expect, thus discovering the future goals and planning actions for achievements (Gutiérrez-Braojos, 2015; Zhang et al., 2015; Wong et al., 2019). Individuals with a high future time orientation tend to be more successful in career and studies (Zimbardo and Boyd, 2008; Gutiérrez-Braojos, 2015), which plays a guiding and incentive role in learning (Lens, 1986; Moreas and Lens, 1991; Lindstrom-Johnson et al., 2016). Thus, the future time orientation contributes to learning, and researchers believe that the higher future time orientation of an individual is, the more he or she can make to engage in tasks and dedicate. In other words, the stronger his or her future time orientation is, the higher commitment to learning will be.

Study of students at university is affected by characteristics when entering and academic and interpersonal integration at the university, and it affects their commitment to goals and university and their external commitment (Tinto, 1997; Schaeper, 2020). Astin (1991) proposed the impact of the Input-Environment-Outcome model (I-E-O model for short) on educational outcomes (Wawrzynski et al., 2012). To improve the quality of higher education, the standard of quality measurement should be transferred from Input, which is originally emphasized, to Output, and emphasis should be attached to the learning process and performance. Elements affecting learning performance of students should be analyzed so that ways for improvement can be figured out. Horstmanshof and Zimitat (2007) argued that engagement has emerged as a key issue in student retention, continued participation in higher education, and examined the relationship between future time orientation and engagement. However, based on expectancy-value theory and social-cognitive theory of motivation, Hsieh (2014) emphasized that learning motivation leads to an increase in engagement behaviors that influence subsequent achievements. According to the discussions and theoretical views from scholars, learning motivation can be regarded as an important mediator between future time orientation and engagement.

In higher education, the most critical factor affecting learning of students is not only to provide students with the experience and knowledge they need while learning, but also to stimulate their positive attitude of autonomous engagement and involvement in learning (Pike et al., 2012; Hsieh, 2014; Suárez et al., 2019). The learning performance of students is an important indicator that shows the quality of teaching at university, but also a key link in the pursuit of excellent development (Beno, 2004). To understand learning evaluation for freshmen, this study aims to adopt the database of student learning process established by the university for Secondary Data Analysis and explores the cross-lagged effect among the future time orientation, self-determination, and learning engagement of freshmen.

## Literature Review

### Self-Determination Theory

Self-determination theory claims three basic psychological needs that influence individual motivation: competence, relatedness, and autonomy (De Bilde et al., 2011). According to the self-determination theory, “autonomy” is the most basic psychological need for each person (Chemolli and Gagné, 2014), but the research with “self” as one of the construction elements for theories is bound to involve the definition of self of individual (Kaplan et al., 2002; De Bilde et al., 2011; Guay and Bureau, 2018). Self-determined motivation is the quality of behavioral operation of people, which refers to choosing, that is, the experience of perceptually internal control (Howard et al., 2017; Ryan and Deci, 2017, 2020). Thus, self-determined motivation indicates that the individual has the opportunity to choose and also has the ability to choose, not external oppression or enhanced sense, which makes him or her do it, it is something that the individual decides (Chemolli and Gagné, 2014; Guay and Bureau, 2018).

In this study, the degree of learning motivation of students measured by the self-determination theory is adopted, which was proposed by Ryan and Deci (2017, 2020), and it is divided into three dimensions of “intrinsic motivation,” “extrinsic motivation,” and “amotivation.” (1) Intrinsic motivation indicates that an individual engages in the activity out of interest on his or her own initiative, and the individual can get pleasure from the activity itself, which belongs to a high degree of autonomy; (2) extrinsic motivation means that external things are an inducement to make individuals engage in various activities, and this motivation exists outside the learning object and is highly interdependent with intrinsic motivation; and (3) amotivation presents that it lacks any motivation, which is associated with quite negative outcomes (Chemolli and Gagné, 2014; Howard et al., 2017; Wigfield et al., 2017).

### Future Time Orientation

Future time orientation is a concept that is used to understand individual behaviors. Zimbardo and Boyd (1999, 2008) believed that future time orientation constitutes our unique psychological background, which has a great impact on minds, emotions, and behaviors of people (De Bilde et al., 2011), and plays a vital role in making individuals understand life experiences and explain behaviors (Gutiérrez-Braojos, 2015; Lindstrom-Johnson et al., 2016). In previous studies, future time orientation was defined as a trait with stable tendency, a cognitive structure, which is flexible and modifiable, or a unidirectional or multidirectional construction, which has attracted much attention from researchers (Zhang et al., 2015; Lindstrom-Johnson et al., 2016; Wong et al., 2019). For instance, De Volder and Lens (1982) viewed that it is a personality trait and divided it into dynamic state and cognition, while Daltrey and Langer (1984) argued that future time orientation is a multi-dimensional construction, such as ductility, consistency, orientation, density, and attitude/emotion (De Bilde et al., 2011). Afterward, Peetsma (2000) regarded future time orientation is a motivational concept and divided it into knowledge, evaluation, and behavioral intention.

Subsequent studies have suggested that people will change their view of time with age and personality course. Husman and Lens (1999) believed that future time orientation is the expected belief about future goals that an individual has. Future goals of students are associated with their current study, work, or academic performance, thus facilitating individuals to have the motivation to learn (Lens et al., 2002; Zhang et al., 2015). The stronger future time orientation of an individual is, the more likely he or she is to develop specific future goals, and the individual is prompted to engage in tasks, make efforts, and plans that help to achieve future goals (Husman and Lens, 1999; De Bilde et al., 2011; Lindstrom-Johnson et al., 2016; Wong et al., 2019). Miller and Brickman (2004) argued that future time orientation is an inducement to achieve future goals and improve willingness of individual learners to engage in autonomous learning (Gutiérrez-Braojos, 2015). Later, Mello and Worrell (2015) summarized future time orientation into several key aspects: first, future time orientation is cognitive, as it originates from minds, and it is also motivated because thinking of individuals about time will prompt his or her to make decisions and perform specific behaviors (Peetsma, 2000; Simons et al., 2004; Wong et al., 2019). Second, the measurement of future time orientation includes ductility, density, consistency, involvement, anticipation, velocity, perceptibility, and value. In other words, when an individual establishes future goals, the more he or she can consider the amount of time and specific plans, the more he or she is concerned about the value of pondering on current tasks and the future. Third, the variability of future time orientation of individuals is derived from learning and social experience in various situations (Miller and Brickman, 2004).

As for the relationship between future time orientation and learning engagement, in the past, Brown and Jones (2004) believed that different views of learners of time orientation would affect their degree of learning engagement and give rise to different learning outcomes (Gutiérrez-Braojos, 2015; Lindstrom-Johnson et al., 2016). Horstmanshof and Zimitat (2007) found that when college students have a better future time orientation, they have a better learning attitude or behavior, such as mastering goals, intending to spend more time reading, adopting deeper learning strategies, and seeking assistance from others when facing difficulties. Future time orientation is positively correlated with learning engagement (King, 1984; De Bilde et al., 2011). Simons et al. (2004) found that future goals can play a significant role in predicting academic performance of students at university, and those intrinsic future goals can lead to greater efforts, autonomous learning, persistence in learning tasks (Gutiérrez-Braojos, 2015), and contributing to more conceptual learning, better performance, and higher persistence in relevant learning activities in the future.

Bembenutty and Karabenick (2003) believed that future time orientation is a belief or tendency of future goals that an individual holds, which can stimulate learning interest, motivation, and behavioral adjustment to pursue future goals (Gutiérrez-Braojos, 2015). Miller and Brickman (2004) put forward the self-adjusting motivation model of future orientation, believing that the motivation of future orientation is the belief system acquired by individuals in the process of socialization, which can form future goals and guide learners to do self-adjustment. High-school students of different degrees have different views when perceiving future time orientation. The higher the learning motivation of students is, the more likely they are to perceive the importance of schoolwork to their future life. Gutiérrez-Braojos (2015) studied and found that if students perceive the value of schoolwork and future development outcomes, it would affect their learning motivation.

### Learning Engagement

Learning engagement is a vital variable in the performance of students (Suárez et al., 2019; Afzali and Izadpanah, 2021). Learning engagement not merely shows learning motivation of students, but also contributes to understanding subsequent behavior and development of students (Afzali and Izadpanah, 2021). The efforts, persistence, concentration, and happiness of those who are highly engaged in learning will produce positive progress and good academic performance (Hsieh, 2014). In contrast, less learning time, abandonment, distraction, sadness, and anxiety of those who are unwilling to engage in are also highly correlated with dropout (Fredricks et al., 2004; Suárez et al., 2019). Learning engagement refers to the time and energy that students devote to educational goals and activities (Afzali and Izadpanah, 2021). Particularly, only through interaction with others can these educational activities be meaningful. Kuh (2003, 2009) defined learning engagement as the process of individual behavior, perception, and thinking in learning of students. The key indicators are the time and energy that students devote to educational goals and activities (Wigfield et al., 2017). In particular, only through interaction with others can these educational activities be meaningful. It follows that despite the words used are different, the meanings are much the same, and all of them emphasize the need for students to devote their mental and physical energy to their study (Hsieh, 2014). For students, high learning engagement of individuals is not merely conducive to their learning outcomes but also can improve the teaching effectiveness for teachers (Afzali and Izadpanah, 2021). Thereby, it is prominently important to pay attention to engagement of students and then consider how to help them devote more to their studies.

Autonomous interest and sense of ability of students in their studies are important components of their academic identity, and academic identity, learning motivation, degree of engagement, and learning accomplishments are related (Abes et al., 2007; Taylor et al., 2014; Guay et al., 2017; Guay and Bureau, 2018). Self-determination can positively affect the learning process, and its behavioral outcomes and learning outcomes are concentrated on learning performance of students (Hummel and Randler, 2012; Hsieh, 2014; Ryan and Deci, 2017, 2020). When learning motivation and active learning of students can play a synergistic role and influence each other, the effect of learning engagement of students will gradually increase.

Self-determination is a process behavior that occurs when students achieve educational or specific goals during learning (Chemolli and Gagné, 2014; Guay and Bureau, 2018), which plays a vital role in future time orientation and learning engagement (Ng et al., 2012; Gagné et al., 2015). For college students with high intrinsic motivation, high future time orientation has higher autonomy than the sense of competence and belonging compared with low future time orientation. However, De Bilde et al. (2011) mentioned that future time orientation plays an effect on intrinsic motivation while students are learning, and students who own future time orientation can adjust their learning behavior and performance in an autonomous way (Gutiérrez-Braojos, 2015). Yorke and Knight (2004) found that learners of self-theory would engage in learning on their own and influence their motivation (Chemolli and Gagné, 2014), impetus, engagement in learning, and even measure the possibility of goal achievement (Ryan and Deci, 2017, 2020; Guay and Bureau, 2018). If the goal is impracticable or impossible to achieve, the motivation to learn will be declined, together with their engagement in learning will be kept down (Sheldon et al., 2017; Howard et al., 2020). Future time orientation can also effectively predict efforts of students in homework. Compared with students with a low future time orientation, students with a high future time orientation are more engaged in learning and more active in learning (Zimbardo and Boyd, 1999; Peetsma, 2000). Future time orientation is positively correlated with learning engagement (Peetsma, 2000; King, 2016), and future time orientation of students can indirectly affect their academic achievement and performance through self-efficacy (Phan, 2014). Based on the above findings, the self-determination theory has a positive correlation between future time orientation and learning engagement and predicts learning engagement through future time orientation. Researchers set out to understand the cross-lagged effect by means of data collected at different times.

**FIGURE 1:**
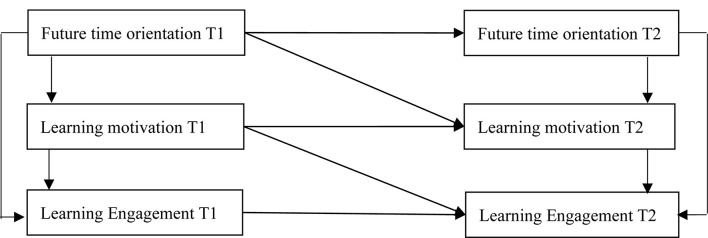
Research framework.

According to the above arguments, based on self-determination theory, this study aims to explore the relationships among future time orientation, learning motivation, and learning engagement in different times. Furthermore, specifically, this study has proposed four hypotheses to verify our research framework, as shown in Figure 1: (1) H1: future time orientation has a positive impact on learning engagement; (2) H2: future time orientation has a positive impact on learning motivation; (3) H3: learning motivation has a positive impact on learning engagement; and (4) H4: there is a cross-lagged effect among future time orientation, learning motivation, and learning engagement.

## Methodology

### Sampling

The data for this study were collected from the database of student learning and performance at some universities in Taiwan, and the data for analysis were obtained by applying to the relevant authorities for data release. In the database, there are questionnaire contents for two stages. For the first stage, there are copies of the questionnaire completed by the freshmen when entering the university, and the testing has been conducted with the questionnaire designed by the past research scale. For the second stage, there are copies of the questionnaire for testing, completed by the freshmen after entering the university and before entering the first summer vacation. To make the research samples representative, the study takes four comprehensive universities as the research objects and limits their university size to the same level, such as the ratio of teachers to students, the number of students, and educational goals. In this study, the data of freshmen enrolled in 2020 were obtained from the database for analysis. In the first stage, 1,000 valid samples were obtained, of which male accounted for 46% and female accounted for 54%; 53% from social sciences and 47% from natural sciences; 36% were first-generation students; and 62% of the students knew their subject. In the second stage, 840 valid samples were obtained, with 45% male and 55% female; 58% from social sciences and 42% from natural sciences; 34% were first-generation students; and 72% of the students knew their subject.

Due to the different types of gender and disciplines, a systematic error might have arisen, bringing the external validity of study into question. Thus, several independent-samples *t*-tests were used to verify whether the male versus female groups and social sciences versus natural sciences groups differed significantly in terms of research dimensions. Due to the different sources of students in the four universities, ANOVA analysis was also conducted on the data to see if there were significant differences. The results indicated that the groups did not significantly differ, so it was deemed appropriate to merge the samples from different gender, disciplines, and universities.

### Measures

Most of the scales in the questionnaire are adopting previous studies and are modified to suit the research context. In future time orientation, we used the scale proposed by Husman and Lens (1999), such as “value,” which refers to the high-value individuals attach to their future goals and indicates the degree of emphasis that one individual attaches to the future goals; “speed,” which refers to the perceived short time for future goals; and “future goals,” which are goals for future orientation. To divide learning motivation into intrinsic motivation, extrinsic motivation, and amotivation, we adopted the scale proposed by Deci and Ryan (1985, 1991) and McLean (2003). Student engagement is evaluated by three scales developed by Pascarella et al. (2013) and Campbell and Cabrera (2014) on the basis of NSSE items completed in the student samples, such as higher order learning, integrative learning, and reflective learning. According to the definition and operation of Laird et al. (2006, 2008) and Pascarella et al. (2013), higher order learning is measured by four items; integrative learning consists of five items; and reflective learning includes two items. All items were measured with a 5-point Likert scale (1, totally disagree; 5, totally agree as shown in Table 1).

**TABLE 1:** Scale of measure variables.

**Construct**	**Variables**	**Items**
Future time orientation	Value	I engage in the present activity is similar to the capacities required to engage in a future task
		Realizing a long-term goal is worth some sacrifices today
		It’s the small steps one takes today which determine one’s future security
	Speed	What will happen in the future is an important consideration in deciding what action to take
		Possible future gains are more important than Immediate gain
		The best choice of action is one which might pay off in the future
	Future goals	It is important to have goals for where one wants to be in five or ten years
		What one does today really does matter much in the long run
		One should be taking steps today to help realize future goals
Learning motivation	Intrinsic motivation	For the pleasure I experience when I feel completely absorbed in university
		For the satisfaction I have when I try to achieve my personal goals in university
		For the interest I have in understanding more about myself
	Extrinsic motivation	Because other people think that it’s a good idea for me to be in university
		Because I don’t want to upset people close to me who want me to be in university
		To satisfy people close to me who want me to get help for my current situation
	Amotivation	Honesty, I really don’t understand what I can get from university
		I wonder what I’m doing in university; actually, I find it boring
		I once had good reasons for going to university, however, now I wonder whether I should quit
Learning engagement	Higher-order learning	Analyzed the basic elements of an idea, experience, or theory, such as examining a particular case or situation in depth and considering its components
		Synthesized and organized ideas, information, or experiences into new, more complex interpretations and relationships
		Made judgments about the value of information, arguments, or methods, such as examining how others gathered and interpreted data and assessing the soundness of their conclusions
		Applied theories or concepts to practical problems or in new situations
	Integrative learning	Worked on a manuscript or project that required integrating ideas or information from various sources
		Included diverse perspectives (different races, religions, genders, and political beliefs, etc.) in class discussions or writing assignments
		Put together ideas or concepts from different courses when completing assignments or during class discussions
		Discussed ideas from your readings or classes with faculty members outside of class
		Discussed ideas from your readings or classes with others outside of class (students, family members, and coworkers, etc.)
	Reflective learning	Examined the strengths and weaknesses of your own views on a topic or issue
		Tried to better understand someone else’s views by imagining how an issue looks from his or her perspective

### Analysis Strategy

This study tested the hypotheses of the research framework and included paths via structural equation modeling. For higher order constructs (future time orientation, learning motivation, and learning engagement), we reduced the number of parameters to be estimated with the method of partial aggregation (Little et al., 2002). This procedure involves averaging the responses of subsets of items that measure a construct. In the measurement model, we first measured all dimensions and provided a rigorous confirmatory factor analysis (CFA) report. Then, we averaged responses of each dimension to serve as indicators for these constructs to simplify the model and enhance the model fitting, because future time orientation, learning motivation, learning engagement were multi-dimensional constructs. Structural model of cross-lagged effect and measurement model were performed using IBM-AMOS statistical program, v. 23.0 for Windows.

## Results

### Evaluation of the Measurement Model

All scales used in this study were found to be reliable, with Cronbach’s α ranging from 0.75 to 0.92. Table 2 shows the reliability of each measurement variable. To examine validity, this study adopted CFA using AMOS 23.0 to verify the construct validity (both convergent and discriminant) of the scales. According to Hair, Black et al. (2010) recommended validity criteria, CFA results show standardized factor loading of higher than 0.5; average variance extracted (AVE) ranges between 0.521 and 0.841; and composite reliability (CR) ranges between 0.719 and 0.955. All three conditions for convergent validity were addressed, and correlation coefficients were all less than the square root of the AVE within one dimension, suggesting that each dimension had well-established discriminant validity.

**TABLE 2:** Measurement properties.

	**1**	**2**	**3**	**4**	**5**	**6**	**7**	**8**	**9**
1. Value	–	0.64	0.10	0.50	0.49	0.22	0.53	0.35	0.52
2. Future goals	0.50	–	0.00	0.41	0.45	0.15	0.45	0.43	0.45
3. Speed	0.21	0.07	–	0.14	0.09	0.42	0.11	−0.11	0.09
4. Intrinsic motivation	0.51	0.33	0.15	–	0.77	0.20	0.71	0.47	0.67
5. Extrinsic motivation	0.46	0.39	0.13	0.52	–	0.20	0.65	0.47	0.60
6. Amotivation	0.37	0.26	0.34	0.26	0.33	–	0.17	−0.07	0.13
7. Higher-order	0.48	0.39	0.11	0.58	0.47	0.22	–	0.60	0.81
8. Integrative	0.33	0.44	0.04	0.38	0.38	0.12	0.51	–	0.62
9. Reflective	0.47	0.40	0.11	0.55	0.44	0.25	0.65	0.48	–
First wave *M*	4.06	3.24	3.51	4.08	3.97	4.20	3.70	3.34	3.67
First wave *SD*	0.74	0.95	0.89	0.69	0.73	0.84	0.71	0.71	0.78
Second wave *M*	3.79	3.46	3.43	4.03	3.91	3.61	3.86	3.44	3.80
Second *SD*	0.77	0.89	0.91	0.74	0.74	1.02	0.70	0.75	0.73
Cronbach’s α	0.87	0.92	0.75	0.79	0.75	0.84	0.84	0.87	0.85
AVE	0.58	0.62	0.55	0.61	0.62	0.66	0.64	0.67	0.62
CR	0.84	0.89	0.88	0.84	0.86	0.91	0.89	0.93	0.87

### Tests for Differences

Before conducting a structural model analysis of the cross-growth effect, this study first examined whether there were differences in all measure variables between T1 and T2 to properly and rigorously perform the cross-lagged effect between T1 and T2. The study aims to explore the changes in the learning state of freshmen after their first year of study, and the paired-sample *t*-test has been adopted for analysis, as shown in Table 3. As for future purpose (t = − 4.26, *p* < 0.001), higher order learning (t = − 3.56, *p* < 0.001), integrative learning (t = − 1.98, *p* < 0.05), and reflective learning (t = − 2.66, *p* < 0.01), the scores of post-test average are higher than those of pre-test average and have reached the significant difference level respectively. Second, Speed (t = 3.17, *p* < 0.01) of future time orientation and amotivation (t = 16.82, *p* < 0.001) of learning motivation are concepts, which are negatively constructed. Notwithstanding that the average score is decreased, students actually have perceived the speed at which time passes faster and have greater confidence in current learning. In regard to the decline in amotivation, it shows that students do not feel that going to university is just a waste of time. University is a palace for all-around development, where they can dig the treasure of knowledge and abundant life experience and become freshmen involved in successful learning.

**TABLE 3:** Paired-sample *t*-test of measure variables.

**Measure variables**	**Time**	***M***	***SD***	**95% CI**		***t***	***Compare***
				**LL**	**UL**		
Value	T1	4.06	0.74	0.26	0.40	9.79***	T1 > T2
	T2	3.79	0.77				
Future goals	T1	3.24	0.95	−0.24	−0.09	−4.26***	T2 > T1
	T2	3.46	0.89				
Speed	T1	3.51	0.89	0.05	0.22	3.17**	T1 > T2
	T2	3.43	0.91				
Intrinsic motivation	T1	4.08	0.69	0.02	0.14	2.53*	T1 > T2
	T2	4.03	0.74				
Extrinsic motivation	T1	3.97	0.73	0.06	0.19	3.83***	T1 > T2
	T2	3.91	0.74				
Amotivation	T1	4.20	0.84	0.63	0.80	16.82***	T1 > T2
	T2	3.61	1.02				
Higher-order	T1	3.70	0.71	−0.17	−0.05	−3.56***	T2 > T1
	T2	3.86	0.7				
Integrative	T1	3.34	0.71	−0.13	0.00	−1.98*	T2 > T1
	T2	3.44	0.75				
Reflective	T1	3.67	0.78	−0.15	−0.02	−2.66**	T2 > T1
	T2	3.80	0.73				

*p < 0.05; **p < 0.01; ***p < 0.001.

### A Cross-Lagged Effect Test

**FIGURE 2:**
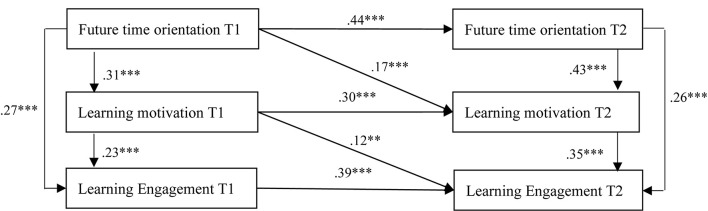
Cross-lagged effect test. ***p < 0.001.

In this study, a cross-lagged effect was detected using SEM in the model via AMOS 23.0. The findings of this study are shown in Figure 2. First, future time orientation T1 (β = 0.27, *p* < 0.001) and T2 (β = 0.26, *p* < 0.001) were positively and significantly related to learning engagement T1 and T2, supporting H1. The standardized path coefficients of future time orientation T1 to learning motivation T1 (β = 0.31, *p* < 0.001) and future time orientation T2 to learning motivation T2 (β = 0.43, *p* < 0.001) are positive and significant. H2 is supported. Furthermore, standardized path coefficients of learning motivation T1 to learning engagement T1 (β = 0.23, *p* < 0.001) and learning motivation T2 to learning engagement T2 (β = 0.35, *p* < 0.001) are also positive and significant, which is supporting H3.

To know the cross-lagged effect of future time orientation, self-determination, and learning engagement, we first analyzed the hypothetical model. In this study, AMOS 23.0 was adopted for structural model analysis. In regard to the inner adaptation of the model, as shown in Figure 2, the path coefficients defined by the model have all reached a significant level. In terms of the autoregression effect, the path coefficient of future time orientation T1 to future time orientation T2 is 0.44 (*p* < 0.001), the standardized path coefficient of learning motivation T1 to learning motivation 2 is 0.30 (*p* < 0.001), and the standardized path coefficient of learning engagement T1 to learning engagement T2 is 0.39 (*p* < 0.001). It follows that the three constructs established in this study are provided with cross-time stability, and the behavioral outcomes of the former wave contribute to predicting the subsequent behaviors. In addition to the autoregression effect, the standardized path coefficient of cross-lagged effect for the future time orientation T1 to learning motivation T2 is 0.17 (*p* < 0.001), and learning motivation T1 to learning engagement T2 is 0.12 (*p* < 0.01), and they have reached a significant level, showing the cross-lagged effect which occurs among future time orientation, learning motivation, and learning engagement of students. Meanwhile, it has also been proved that future time orientation of freshmen could affect their learning engagement by learning motivation, which is supporting H4.

## Conclusion

### Discussion

The research findings show that self-determination theory refers to a process behavior for achieving educational or specific goals while students are learning (Chemolli and Gagné, 2014), which plays a vital role in future time orientation and learning engagement (Taylor et al., 2014). Future time orientation can also effectively predict efforts of students in homework. Compared to students with a low future time orientation, students with a high future time orientation are more engaged in learning and perform more actively in learning (Peetsma, 2000; De Bilde et al., 2011; Visser and Hirsch, 2014; Gutiérrez-Braojos, 2015). Simons et al. (2004) indicated that future goals can play an important role in predicting academic performance of students at university (Lindstrom-Johnson et al., 2016), and those intrinsic future goals can lead to greater efforts, autonomous learning, and persistence in learning tasks and give rise to more conceptual learning, better performance, and higher persistence in relevant learning activities in the future (De Bilde et al., 2011; Guay and Bureau, 2018). Self-determination plays a positive effect on the learning process, of which the behavioral outcomes and learning outcomes are concentrated on learning performance of students (Hummel and Randler, 2012; Chemolli and Gagné, 2014; Gutiérrez-Braojos, 2015; Howard et al., 2017, 2020). When people are more intrinsically motivated to engage in a certain activity, they are more likely to be engaged in learning and they can engage in the activity for a longer time (Ryan and Deci, 2017, 2020).

The findings indicate that there is a partially and significantly positive correlation among the elements of future time orientation, self-determination, and learning engagement of students. When students own a high future time orientation or high learning motivation, they are provided with higher learning engagement (Hsieh, 2014; Lindstrom-Johnson et al., 2016). This is similar to the results from previous studies, Zimbardo and Boyd (1999), Peetsma (2000), Husman and Shell (2008), and De Bilde et al. (2011) believed that future time orientation or learning motivation is positively correlated with learning engagement. When learners can perceive that what they have learned at university provides value for their future life or they are in need of school knowledge, they will be more engaged in learning (Visser and Hirsch, 2014; Gutiérrez-Braojos, 2015). Thus, the higher degree of future time orientation students owns, the more they can establish their own future goals (De Bilde et al., 2011) and reflect on the advantages and disadvantages of their existing behaviors to prepare for future goals, thus inducing their learning engagement behavior.

Furthermore, there is a cross-lagged effect among future time orientation, self-determination, and learning engagement of students, and it can be proved that future time orientation of students can affect their future learning engagement via self-determination. Students with a future time orientation can consider future task outcomes beforehand, which influences their motivation for future goals (Taylor et al., 2014), and then engage in learning activities that are beneficial to the future (Carstensen, 2006). As Miller and Brickman (2004) believed that the importance and value of future time orientation lie in its guidance to make learners do self-adjustment with the incentive of achieving future goals (De Bilde et al., 2011). People with a future time orientation view time as a kind of resource have enough time to imagine their desired outcomes, ponder on alternative strategies, and gather a wide range of information (Pennington and Roese, 2003). That is, self-determination positively affects the learning process, and behavioral outcomes and learning outcomes are concentrated on learning performance of students (Hummel and Randler, 2012). Skinner et al. (2008) argued that engagement is an integral part of the motivation architecture and that the two influence each other. In an activity, an individual has motivation but is not necessarily actively engaged. For engagement, motivation is a necessary but not sufficient condition. Thus, the degree of learning engagement can be applied to know whether an individual has learning motivation.

Besides, it is found in the study that the cross-lagged effects of future time orientation T1 to learning motivation T2 and learning motivation T1 to learning engagement T2 are not as intense as other cross-lagged paths, but the effects are still positive and significant. The reason maybe that the perception degree of future time orientation and learning motivation for freshmen in T1 may require more time to adapt to the learning environment, and there maybe a process effect formed among the three dimensions, which make the coefficients of the two paths significant but not as strong as the effect found in each time point. Despite the mediating effect of learning motivation has not been examined, it can be found from the structural model that the indirect effects in T1 and T2 are, respectively, 0.0713 and 0.1505, indicating that there are partial mediating effects on learning motivation in T1 and T2. Our study also contributes to the research gap in the application of self-determination and expectancy-value theory to learning motivation (Horstmanshof and Zimitat, 2007; Hsieh, 2014). These findings indicated that freshmen who reported a greater sense of future time orientation having experienced, a higher degree of intrinsic motivation; specific future goals for achieving; a high level of learning engagement.

### Implications

The study shows that only through self-determination can the future time orientation be enough to influence the learning engagement of freshmen. In regard to the viewpoint of Chinese education, Chinese education starts from future time orientation, and learners usually base their learning goals on long-term future achievements. However, for the sake of achievements, the state of learning motivation for individuals during the process will affect subsequent achievements and performance of students and constant engagement in campus activities. Freshmen are suggested to blend in the future time orientation before entering the sophomore year, and it is suggested to know how students view the future, discuss the future with freshmen, strengthen learning motivation, and maintain and stimulate the inner thinking process of student behavior to achieve a specific goal, which will affect the performance of learning engagement, but also gain more campus experience to achieve future goals.

The findings state that students with high learning motivation will increase their degree of learning engagement. This study suggests that when universities take student learning performance as a direction for school running, the important aspects of effective learning should be taken into consideration, and it is necessary to know how to stimulate learning motivation of students (Tella, 2007). When traditional writing, reading, and literacy may fail to meet the demand of some native generation students, teachers should know congenital traits of students, change teaching patterns, and trigger students to engage in learning activities, maintain learning activities, and improve their intrinsic motivation via multiple orientations.

It is found that despite future time orientation and learning motivation of freshmen are waning, their learning engagement increases along with the promotion of various programs. Universities have the responsibility to create a more supportive system, such as reducing barriers to higher education institutions to assist students in engaging in effective learning activities (Greene et al., 2008). In addition to promoting the current programs, semi-structured interviews for partial students are available. Based on the view centering on learning of students, students’ understanding of the existing campus tutoring mechanism needs to be figured out, and in terms of activities for promotion, whether there are innovative activities corresponding to students that are beneficial to funds injection from universities and make relevant quality indices effective needs to be resolved.

### Research Limitations and Directions for Future Studies

The research results contribute to the literature on SDT and student learning engagement; nevertheless, some limitations still exist and represent further research directions. First, climacteric of teenagers is a significant topic for exploring time orientation (Bitti et al., 2015), which is a process from adolescence to adulthood. The future composition changes and develops with age (McInerney, 2004). In this study, survey data of the second wave collected from a number of freshmen were taken as the basis for analysis. Since there is an issue of sample loss, the learning process data of these students are constantly collected for subsequent research, and investigations of the learning process at different periods are conducted respectively before the sophomore and junior summer vacation and before the graduation of senior year so that changes in the passing time and the situation of learning engagement can be figured out. Besides, a variety of learning norm data are established to provide immediate feedback for subsequent questionnaire takers and even cross-level analysis.

Second, although the research framework was constructed with reference to SDT in this study, and important learning theories can be derived from the research results, other motivation theories, such as social cognitive career theory, self-efficacy theory, and emotion cognition theory, still apply to explain how to trigger cross-lagged learning in freshmen. Thus, it is suggested that future research can utilize different theoretical models to identify relevant psychological dimensions influencing learning engagement of students.

Third, due to restrictions of time and space, only few universities were sampled in this study, with 840 valid questionnaires in total. Future research could explore and compare other groups, in addition to expanding the number of samples and improving the research representativeness, to provide additional insights relevant to future time orientation literature and higher education.

## Data Availability Statement

The original contributions presented in the study are included in the article/supplementary material, further inquiries can be directed to the corresponding author.

## Ethics Statement

The studies involving human participants were reviewed and approved by University of Taipei. The patients/participants provided their written informed consent to participate in this study.

## Author Contributions

MP and ZZ contributed to the ideas of educational research, collection of data, and empirical analysis. MP contributed to the data analysis, design of research methods, and tables. ZZ participated in developing a research design, writing, and interpreting the analysis. All authors contributed to the literature review and conclusions.

## Conflict of Interest

The authors declare that the research was conducted in the absence of any commercial or financial relationships that could be construed as a potential conflict of interest.

## Publisher’s Note

All claims expressed in this article are solely those of the authors and do not necessarily represent those of their affiliated organizations, or those of the publisher, the editors and the reviewers. Any product that may be evaluated in this article, or claim that may be made by its manufacturer, is not guaranteed or endorsed by the publisher.

## References

[B1] Abes E. S. Jones S. R. McEwen M. K. (2007). Reconceptualizing the model of multiple dimensions of identity: the role of meaning-making capacity in the construction of multiple identities. *J. Coll. Stud. Dev.* 48 1–22. 10.1353/csd.2007.0000 34409987

[B2] Afzali Z. Izadpanah S. (2021). The effect of the flipped classroom model on Iranian English foreign language learners: engagement and motivation in English language grammar. *Cogent Educ.* 8:1870801. 10.1080/2331186X.2020.1870801

[B3] Astin A. W. (1991). *Assessment for Excellence: The Philosophy and Practice of Assessment and Evaluation in Higher Education.* New York, NY: American Council on Education.

[B4] Bembenutty H. Karabenick S. A. (2003). “Academic delay of gratification, future goals, and self-regulated learning,” in *Paper Presented at the annual meeting of the American Educational Research Association (ERIC Document Reproduction Service No. ED479131)*, Chicago.

[B5] Beno B. A. (2004). “The role of student learning outcomes in accreditation quality review,” in *Developing and Implementing Assessment of Student Learning Outcomes*, eds Serban A. M. Friedlander J. (San Francisco, CA: Jossey-Bass), 65–72. 10.1002/cc.155

[B6] Bitti P. Zambianchi M. Bitner J. (2015). “Time perspective and positive aging,” in *Time Perspective Theory: Review, Research and Application*, eds Stolarski M. Nicolas F. Wessel V. B. (New York, NY: Springer), 437–450. 10.1007/978-3-319-07368-2_28

[B7] Black D. S. Sussman S. Unger J. B. (2010). A further look at the intergenerational transmission of violence: witnessing interparental violence in emerging adulthood. *J. Interpers. Violence* 25 1022–1042. 10.1177/0886260509340539 19801446 PMC3705927

[B8] Brown W. Jones J. (2004). The substance of things hoped for: a study of the future orientation, minority status perceptions, academic engagement, and academic performance of black high school students. *J. Black Psychol.* 30 248–273. 10.1177/0095798403260727

[B9] Campbell C. M. Cabrera A. F. (2014). Making the mark: are grades and deep learning related? *Res. High. Educ.* 55 494–507. 10.1007/s11162-013-9323-6

[B10] Carstensen L. L. (2006). The influence of a sense of time on human development. *Science* 312 1913–1915. 10.1126/science.1127488 16809530 PMC2790864

[B11] Chemolli E. Gagné M. (2014). Evidence against the continuum structure underlying motivation measures derived from self-determination theory. *Psychol. Assess.* 26:575. 10.1037/a0036212 24611788

[B12] Daltrey M. Langer P. (1984). Development and evaluation of a measure of future time perspective. *Percep. Motor Skills* 58 719–725. 10.2466/pms.1984.58.3.719

[B13] De Bilde J. Vansteenkiste M. Lens W. (2011). Understanding the association between future time perspective and self-regulated learning through the lens of self-determination theory. *Learn. Instr.* 21 332–344. 10.1016/j.learninstruc.2010.03.002

[B14] De Volder M. L. Lens W. (1982). Academic achievement and future time perspective as a cognitive-motivational concept. *J. Pers. Soc. Psychol.* 42 566–571. 10.1037/0022-3514.42.3.566

[B15] DeBerard M. S. Spielmans G. I. Julka D. C. (2004). Predictors of academic achievement and retention among college freshmen: a longitudinal study. *Coll. Stud. J.* 38 66–80.

[B16] Deci E. L. Ryan R. M. (1985). *Intrinsic Motivation and Self-Determination in Human Behavior.* New York, NY: Plenum. 10.1007/978-1-4899-2271-7

[B17] Deci E. L. Ryan R. M. (1991). “A motivational approach to self: integration in personality,” in *Nebraska Symposium on Motivation: Perspectives on Motivation*, ed. Dienstbier R. (Lincoln: University of Nebraska), 237–288. 2130258

[B18] Fredricks J. A. Blumenfeld P. C. Paris A. H. (2004). School engagement: potential of the concept, state of evidence. *Rev. Educ. Res.* 74 59–109. 10.3102/00346543074001059

[B19] Gagné M. Forest J. Vansteenkiste M. Crevier-Braud L. Van Den Broeck A. Aspeli A. K. (2015). The multidimensional work motivation scale: validation evidence in seven languages and nine countries. *Eur. J. Work Org. Psychol.* 24 178–196. 10.1080/1359432X.2013.877892

[B20] Gjesme T. (1983). Worry and emotionality components of test anxiety in relation to situational and personality determinants. *Psychol. Rep.* 52 267–280. 10.2466/pr0.1983.52.1.267 6844496

[B21] Greene T. G. Marti C. McClenney K. (2008). The effort–outcome gap: differences for African American and hispanic community college students in student engagement and academic achievement. *J. High. Educ.* 79 513–539. 10.1080/00221546.2008.11772115

[B22] Guay F. Bureau J. S. (2018). Motivation at school: differentiation between and within school subjects matters in the prediction of academic achievement. *Contemp. Educ. Psychol.* 54 42–54. 10.1016/j.cedpsych.2018.05.004

[B23] Guay F. Denault A. S. Renauld S. (2017). School attachment and relatedness with parents, friends and teachers as predictors of students’ intrinsic and identified regulation. *Contemp. Educ. Psychol.* 51 416–428. 10.1016/j.cedpsych.2017.10.001

[B24] Gutiérrez-Braojos C. (2015). Future time orientation and learning conceptions: effects on metacognitive strategies, self-efficacy beliefs, study effort and academic achievement. *Educ. Psychol.* 35 192–212. 10.1080/01443410.2013.858101

[B25] Horstmanshof L. Zimitat C. (2007). Future time orientation predicts academic engagement among first-year university students. *Br. J. Educ.* 77 703–718. 10.1348/000709906X160778 17908382

[B26] Howard J. L. Gagné M. Bureau J. S. (2017). Testing a continuum structure of self-determined motivation: a meta-analysis. *Psychol. Bull.* 143 1346–1377. 10.1037/bul0000125 29048175

[B27] Howard J. L. Gagné M. Van den Broeck A. Guay F. Chatzisarantis N. Ntoumanis N. (2020). A review and empirical comparison of motivation scoring methods: an application to self-determination theory. *Motiv. Emot.* 44 534–548. 10.1007/s11031-020-09831-9

[B28] Hsieh T. L. (2014). Motivation matters? The relationship among different types of learning motivation, engagement behaviors and learning outcomes of undergraduate students in Taiwan. *High. Educ.* 68 417–433. 10.1007/s10734-014-9720-6

[B29] Hummel E. Randler C. (2012). Living animals in the classroom: a meta-analysis on learning outcome and a treatment–control study focusing on knowledge and motivation. *J. Sci. Educ. Technol.* 21 95–105. 10.1007/s10956-011-9285-4

[B30] Husman J. Lens J. (1999). The role of the future in student motivation. *Educ. Psychol.* 34 113–125. 10.1207/s15326985ep3402_4

[B31] Husman J. Shell D. F. (2008). Beliefs and perceptions about the future: a measurement of future time perspective. *Learn. Individ. Differ.* 18 166–175. 10.1016/j.lindif.2007.08.001

[B32] Kaplan A. Middleton M. J. Urdan T. Midgley C. (2002). “Achievement goals and goal structures,” in *Goals, Goal Structures, and Patterns of Adaptive Learning*, ed. Midgley C. (Mahwah NJ: Lawrence Erlbaum Associates Publishers), 21–53.

[B33] King D. (1984). *Fiscal Tiers: The Economics of Multi-Level Government.* London: George Allen and Unwin.

[B34] King R. B. (2016). Does your approach to time matter for your learning? The role of time perspectives on engagement and achievement. *Int. J. Exp. Educ. Psychol..* 36 1264–1284. 10.1080/01443410.2015.1045835

[B35] Kuh G. D. (2003). What we’re learning about student engagement from NSSE. *Change* 35 24–32. 10.1080/00091380309604090

[B36] Kuh G. D. (2009). The national survey of student engagement: conceptual and empirical foundations. *N. Dir. Inst. Res.* 141 5–20. 10.1002/ir.283

[B37] Laird T. F. N. Niskodé A. S. Kuh G. D. (2006). “General education courses and the promotion of essential learning outcomes,” in *Paper Presented at the Annual Meeting of the Association for the Study of Higher Education*, Anaheim, CA.

[B38] Laird T. F. N. Shoup R. Kuh G. D. Schwarz M. J. (2008). The effects of discipline on deep approaches to student learning and college outcomes. *Res. High. Educ.* 49 469–494. 10.1007/s11162-008-9088-5

[B39] Lens W. (1986). “Future time perspective: a cognitive motivational concept,” in *Frontiers of Motivational Psychology*, eds Brown D. R. Veroff J. (New York, NY: Springer-Verlag), 173–190. 10.1007/978-1-4684-6341-5_10

[B40] Lens W. Simons J. Dewitte S. (2002). “From duty to desire: the role of students’ future time perspective and instrumentality perceptions for study motivation and self-regulation,” in *Academic Motivation of Adolescents*, eds Pajares F. Urdan T. (Greenwich, CT: Information Age Publication), 221–245.

[B41] Levitt S. D. List J. A. Neckermann S. Sadoff S. (2016). The behavioralist goes to school: leveraging behavioral economics to improve educational performance. *Am. Econ. J.* 8 183–219. 10.1257/pol.20130358

[B42] Lindstrom-Johnson S. R. Pas E. Bradshaw C. P. (2016). Understanding the association between school climate and future orientation. *J. Youth Adolesc.* 45 1575–1586. 10.1007/s10964-015-0321-1 26104381

[B43] Little T. D. Cunningham W. A. Shahar G. Widaman K. F. (2002). To parcel or not to parcel: exploring the question, weighing the merits. *Struct. Equ. Modeling* 9 151–173. 10.1207/S15328007SEM0902_1

[B44] McInerney D. M. (2004). A discussion for future time perspective. *Educ. Psychol. Rev.* 16 141–151. 10.1023/B:EDPR.0000026610.18125.a3

[B45] McLean A. (2003). *The Motivated School.* Lodon: Sage.

[B46] Mello Z. R. Worrell F. C. (2015). “The past, the present, and the future: a conceptual model of time perspective in adolescence,” in *Time Perspective Theory: Review, Research and Application*, eds Stolarski M. Nicolas F. Wessel V. B. (New York, NY: Springer), 115–130. 10.1007/978-3-319-07368-2_7

[B47] Miller R. B. Brickman S. A. (2004). A model of future oriented motivation and self-regulation. *Educ. Psychol. Rev.* 16 9–33. 10.1023/B:EDPR.0000012343.96370.39

[B48] Moreas A. M. Lens W. (1991). The motivational meaning of the individual future time perspective. *Learn. Instr.* 2 135–149.

[B49] Ng J. Y. Y. Ntoumanis N. Thogersen-Ntoumani C. Deci E. L. Ryan R. M. Duda J. L. (2012). Self-determination theory applied to health contexts: a metaanalysis. *Perspect. Psychol. Sci.* 7 325–340. 10.1177/1745691612447309 26168470

[B50] Pascarella E. T. Wang J. S. Trolian T. L. Blaich C. (2013). How the instructional and learning environments of liberal arts colleges enhance cognitive development. *High. Educ.* 66 569–583. 10.1007/s10734-013-9622-z

[B51] Peetsma T. T. D. (2000). Future time perspective as a predictor of school investment. *Scand. J. Educ. Res.* 44 177–192. 10.1080/713696667

[B52] Pennington G. L. Roese N. J. (2003). Regulatory focus and temporal distance. *J. Exp. Soc. Psychol.* 39 563–576. 10.1016/S0022-1031(03)00058-1

[B53] Phan H. P. (2014). Situating psychosocial and motivational factors in learning contexts. *Education* 4 53–66. 10.5923/j.edu.20140403.01 22499009

[B54] Pike G. R. Smart J. C. Ethington C. A. (2012). The mediating effects of student engagement on the relationships between academic disciplines and learning outcomes: an extension of Holland’s theory. *Res. High. Educ.* 53 550–575. 10.1007/s11162-011-9239-y

[B55] Ryan R. M. Deci E. L. (2017). *Self-Determination Theory: Basic Psychological Needs in Motivation, Development, and Wellness.* New York, NY: Guilford Publications. 10.1521/978.14625/28806

[B56] Ryan R. M. Deci E. L. (2020). Intrinsic and extrinsic motivation from a self-determination theory perspective: definitions, theory, practices, and future directions. *Contemp. Educ. Psychol.* 61:101860. 10.1016/j.cedpsych.2020.101860

[B57] Schaeper H. (2020). The first year in higher education: the role of individual factors and the learning environment for academic integration. *High. Educ.* 79 95–110. 10.1007/s10734-019-00398-0

[B58] Sheldon K. M. Osin E. N. Gordeeva T. O. Suchkov D. D. Sychev O. A. (2017). Evaluating the dimensionality of self-determination theory’s relative autonomy continuum. *Pers. Soc. Psychol. Bull.* 43 1215–1238. 10.1177/0146167217711915 28903685

[B59] Simons J. Vansteenkiste M. Lens W. Lacante M. (2004). Placing motivation and future time perspective theory in a temporal perspective. *Educ. Psychol. Rev.* 16 121–139. 10.1023/B:EDPR.0000026609.94841.2f

[B60] Skinner E. Furrer C. Marchand G. Kindermann T. (2008). Engagement and disaffection in the classroom: part of a larger motivational dynamic? *J. Educ. Psychol.* 100 765–781. 10.1037/a0012840

[B61] Suárez N. Regueiro B. Estévez I. del Mar Ferradás M. Guisande M. A. Rodríguez S. (2019). Individual precursors of student homework behavioral engagement: the role of intrinsic motivation, perceived homework utility and homework attitude. *Front. Psychol.* 10:941. 10.3389/fpsyg.2019.00941 31080431 PMC6497780

[B62] Taylor G. Jungert T. Mageau G. A. Schattke K. Dedic H. Rosenfield S. (2014). A self-determination theory approach to predicting school achievement overtime: the unique role of intrinsic motivation. *Contemp. Educ. Psychol.* 39 342–358. 10.1016/j.cedpsych.2014.08.002

[B63] Tella A. (2007). The impact of motivation on student’s academic achievement and learning outcomes in mathematics among secondary school students in Nigeria. *Eur. J. Math. Sci. Technol. Educ.* 3 149–156. 10.12973/ejmste/75390

[B64] Tinto V. (1997). Classrooms as communities: exploring the educational character of student persistence. *J. High. Educ.* 68 599–623. 10.1080/00221546.1997.11779003

[B65] Visser P. L. Hirsch J. K. (2014). Health behaviors among college students: the influence of future time perspective and basic psychological need satisfaction. *Health Psychol. Behav. Med.* 2 88–99. 10.1080/21642850.2013.872992 25750770 PMC4346033

[B66] Wawrzynski M. R. Heck A. M. Remley C. T. (2012). Student engagement in South African higher education. *J. Coll. Stud. Dev.* 53 106–123. 10.1353/csd.2012.0007 34409987

[B67] Wigfield A. Eccles J. S. (eds) (2002). *Development of Achievement Motivation.* San Diego, CA: Academic Press, 91–120. 10.1016/B978-012750053-9/50006-1

[B68] Wigfield A. Rosenzweig E. Eccles J. (2017). *Achievement Values. in Handbook of Competence and Motivation: Theory and Application.* New York, NY: Guilford Press, 116–134.

[B69] Wong T. K. Parent A. M. Konishi C. (2019). Feeling connected: the roles of student-teacher relationships and sense of school belonging on future orientation. *Int. J. Educ. Res.* 94 150–157. 10.1016/j.ijer.2019.01.008

[B70] Yorke M. Knight P. (2004). Self theories: some implications for teaching and learning in higher education. *Stud. High. Educ.* 29 25–37. 10.1080/1234567032000164859

[B71] Zhang W. Chen L. Yu F. Wang S. Nurmi J. E. (2015). Hopes and fears for the future among Chinese adolescents. *J. Res. Adolesc.* 25 622–629. 10.1111/jora.12166

[B72] Zimbardo P. G. Boyd J. (1999). Putting time in perspective: a valid, reliable individual- differences metric. *J. Pers. Soc. Psychol.* 77 1271–1288. 10.1037/0022-3514.77.6.1271

[B73] Zimbardo P. G. Boyd J. (2008). *The Time Paradox: The New Psychology of Time that Will Change Your Life.* New York, NY: Free Press.

